# Efficient Decoding of 2D Structured Illumination with Linear Phase Stepping in X-Ray Phase Contrast and Dark-Field Imaging

**DOI:** 10.1371/journal.pone.0087127

**Published:** 2014-01-28

**Authors:** Katherine J. Harmon, Eric E. Bennett, Andrew A. Gomella, Han Wen

**Affiliations:** Imaging Physics Laboratory, Biochemistry and Biophysics Center, National Heart, Lung and Blood Institute, National Institutes of Health, Bethesda, Maryland, United States of America; Mayo Clinic College of Medicine, United States of America

## Abstract

The ability to map the phase distribution and lateral coherence of an x-ray wavefront offers the potential for imaging the human body through phase contrast, without the need to deposit significant radiation energy. The classic means to achieve this goal is structured illumination, in which a periodic intensity modulation is introduced into the image, and changes in the phase distribution of the wavefront are detected as distortions of the modulation pattern. Two-dimensional periodic patterns are needed to fully characterize a transverse wavefront. Traditionally, the information in a 2D pattern is retrieved at high resolution by acquiring multiple images while shifting the pattern over a 2D matrix of positions. Here we describe a method to decode 2D periodic patterns with single-axis phase stepping, without either a loss of information or increasing the number of sampling steps. The method is created to reduce the instrumentation complexity of high-resolution 2D wavefront sensing in general. It is demonstrated with motionless electromagnetic phase stepping and a flexible processing algorithm in x-ray dark-field and phase contrast imaging.

## Introduction

By introducing a periodic modulation in the intensity distribution of a wavefront, perturbations in the phase and lateral coherence of the wavefront become visible as distortions of the pattern and attenuation of its amplitude. Early examples are the Hartmann screen [1] and the Shack-Hartmann lens array [2]. Originally proposed for astronomical telescopes, various embodiments now span diverse areas such as adaptive-wavefront two photon microscopes [3], free-electron x-ray lasers [4], and diagnostic exams of the human eye [5].

In x-ray imaging, the idea is being intensively pursued in two converging efforts: phase contrast imaging and scatter imaging/correction [6]–[13]. X-ray phase contrast imaging can detect weakly absorbing structures by the variation of the refractive index without the need for significant radiation absorption [14], while scatter imaging can characterize unresolved microscopic structures by their diffraction of the x-rays [10], [11], [15]. Scattering reduces the lateral coherence of the wavefront, which is detectable as a drop in the visibility of the modulation pattern. In the human body, x-ray scattering causes a diffuse background, or “fog”, that degrades image quality in diagnostic exams. The “fog” can be quantified and removed through grid modulation of the beam [12], [13].

The intensity modulation is imposed either with simple geometric shadowing of an absorption mask or grid [10], [12], [13], or several forms of wave interferometry for high density patterns [7]–[9], [16]. Some means of measuring the position and amplitude of the fringes of the periodic pattern is needed to retrieve the phase and scattering information. In a single image, the measurements can be done directly in real space [2], [17], or very rapidly with a Fourier transformation method [10], [18]–[21]. The tradeoff of these approaches is that the density of the measurement is limited by the spacing between the fringes. Thus, the phase stepping method was created to provide measurements at the full resolution of the detector. Here, a series of images are taken while the pattern is shifted incrementally, such that the fringes sweep over every point in the image [22]. Every point undergoes at least one complete cycle of intensity oscillation, allowing the fringe position (phase) and amplitude to be measured for that point. Practically, this is realized by either physically scanning a grating or grid [8], [9], [22]–[24], or electromagnetically moving the x-ray source spot to cause a relative displacement between the projection of the sample and the underlying periodic pattern (electromagnetic phase stepping) [25].

Currently, phase stepping of 2D modulations is thought to require raster scanning in two directions [26]–[31]. For example, a rectangular grid pattern aligned with the X and Y axis is demodulated by raster scanning in those directions over a 2D matrix of positions [26]. This adds complexity to mechanical scanning systems. The electromagnetic phase stepping method is also suitable for linear scanning due to the design of common x-ray tubes. Therefore, we present a method of linear phase stepping for retrieving all the information in a 2D periodic pattern.

## Materials and Methods

*Ethics Statement*: The *ex vivo* mouse specimen imaging study was performed under a National Heart, Lung and Blood Institute Animal Care and Use Committee approved protocol.

**Figure 1:**
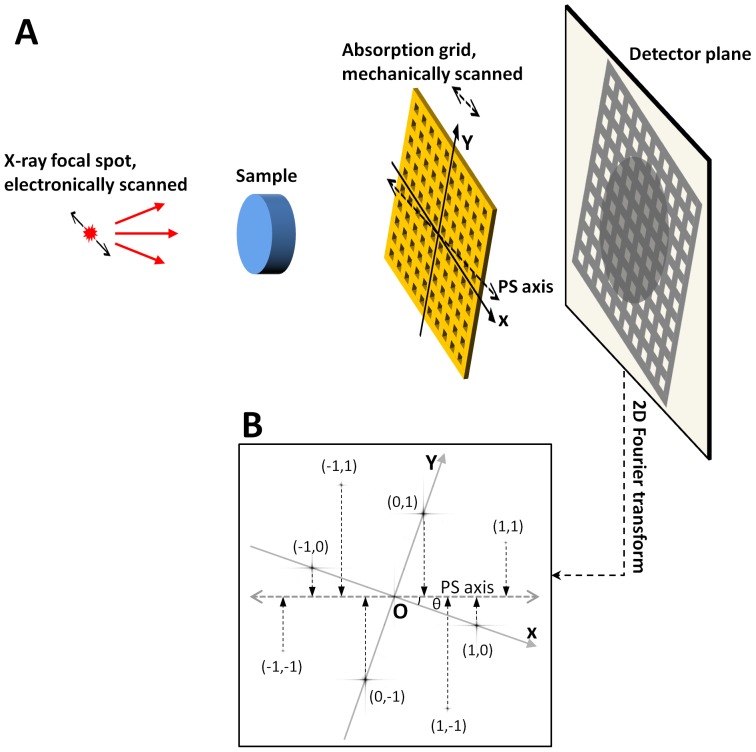
Linear phase stepping in x-ray imaging with a square-lattice absorption grid. (**A**) The projection of the grid imposes a 2D lattice pattern onto the transmitted wavefront. X-ray refractive bending and scattering in the sample cause the pattern to deform and fade, respectively. Linear phase stepping is used to measure these effects at high spatial resolution. The X axis of the grid is at an angle *θ* from the phase stepping axis (PS axis). In mechanical phase stepping, the grid is physically moved along the PS axis. In electromagnetic phase stepping, the focal spot of the x-ray cone beam is scanned with an electromagnetic field, also along the PS axis. (**B**) The algorithm of linear phase stepping is illustrated in the 2D Fourier space (reciprocal space), where the Fourier transformation of the image contains a 3×3 matrix of peaks marked as (0, 1), (1, 1) etc, within the detector's resolution. They correspond to the harmonic sinusoidal waves in real space that make up the 2D lattice pattern. During phase stepping, the phase shift of each harmonic is proportional to its projection onto the PS axis in the reciprocal space. By setting the angle *θ* to 18.4°, the projections are all separated. They can then be determined by a minimum of 9 sample steps along the PS axis in real space. Once the component harmonics are known, both the deformation and the attenuation of the 2D grid pattern are determined at the full resolution of the detector.

The method can be illustrated with the example of a square grid, although applicable to all 2D periodic patterns. In the experimental setup in Fig. 1A, the projection of an absorption grid creates the 2D pattern on the image plane. The pattern is a sum of harmonic sinusoidal waves, including the one for the vertical fringes *H*_x_(**r**)cos[2*πx*/*P*+*φ*_x_(**r**)+*φ*_xj_(**r**)], for the horizontal fringes *H*_y_(**r**)cos[2*πy*/*P*+*φ*_y_(**r**)+*φ*_yj_(**r**)], diagonal mixtures of the two *H*_1,1_(**r**)cos[2*πx*/*P*+2*πy*/*P*+*φ*_1,1_(**r**)+*φ*_1,1,j_(**r**)] and *H*_1,−1_(**r**)cos[2*πx*/*P*+2*πy*/*P*+*φ*_1,−1_(**r**)+*φ*_1,−1,j_(**r**)], and possibly higher order harmonics. Here *P* is the size of the squares, the *H*(**r**)s are the amplitudes of the harmonic oscillations, the *φ*(**r**)s are the phase distortions of the harmonics in the X, Y and diagonal directions, and the *φ*_j_(**r**)s are applied instrumental phase shifts in the phase stepping process in the *j*th step.

The desired information is the phase distortions *φ*(**r**) of the various harmonics and the associated fringe amplitudes *H*(**r**). The phase distortions *φ*(**r**) measure the deformation of the harmonic waves, which are proportional to the gradients in the wavefront phase distribution. In x-ray imaging they arise from refractive bending of the rays in the sample. The fringe amplitudes *H*(**r**) are attenuated by x-ray scattering along the directions of the harmonics, providing a window into the underlying micro structures and their directional orders.

Decoding by linear phase stepping works by incrementally shifting the pattern along an oblique axis relative to the fringe lines. This is graphically illustrated in the 2D Fourier space (reciprocal space) in Fig. 1B. The 2D Fourier transformation of an image has distinct peaks corresponding to the various harmonics. In linear phase stepping, the applied phase shift to each harmonic peak is proportional to its projection onto the oblique axis. By choosing the axis such that the projections of all peaks are fully separated with no two projections coinciding, the 2D harmonics are mapped onto a single axis as different frequencies of oscillation. They can then be retrieved by sampling in real space a series of phase stepped images along the axis. The minimum number of sampling steps is equal to the number of 2D harmonics that are within the detector's resolution. In the example of Fig. 1B, 9 harmonic peaks are captured in the reciprocal space, and hence at least 9 steps are required. The general case of a square matrix of (2*N*+1)×(2*N*+1) harmonic peaks is described in Discussion. Practically, more than the minimum steps are often sampled to improve the signal-to-noise ratio of the results.

Various instrumental instabilities may cause both temporal and spatial variations in the phase steps. Therefore, a retrospective image processing algorithm was created to determine the distribution of the applied phase shifts from the images themselves without assuming *a priori* knowledge [32]. The algorithm is presented in detail in ref. [32] and summarized here. Mathematically, the *j*th image of a phase stepped series can be expressed as
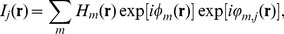
(1)with *H_m_*(**r**) and *φ_m_*(**r**) being the amplitude and phase of the *m*th harmonic of the 2D pattern which we aim to recover, and *φ_m_*_,*j*_(**r**) being the applied instrumental phase shift in the phase stepping process. If *φ_m_*_,*j*_(**r**) is known for all phase steps and harmonics and all locations, then *H_m_*(**r**) and *φ_m_*(**r**) can be determined on a point-by-point basis with a least-squares fitting procedure. The requisite condition is that the number of phase steps is no less than the number of harmonics we need to solve. To retrospectively determine the applied phase shifts, the value for the first image of the series (*j* = 0) is set to zero. The phase increments of the other images relative to the 1st are determined by the Fourier transformation method [18], under the assumption that the grid or grating periods are either uniform or vary smoothly in space. Specifically, in the 2D Fourier space the positions of the harmonic peaks are determined either from the known period of the grid or grating or from the intensity maxima in the Fourier space; a circular window centered around a peak is selected, making sure that its radius is less than half the distance to the nearest-neighbor peak; the window is shifted back to the origin of the Fourier space and inverse Fourier transformed to yield a low-resolution, complex image *h_m_*_,*j*_(**r**) for the *m*th harmonic and *j*th phase step; the phase differential map of *h_m_*_,*j*_(**r**)/*h_m_*_,0_(**r**) is then the applied phase shift of the *j*th phase step. It is interpolated back to the full resolution of the detector, and fed into a least-squares fitting routine to determine the phase and amplitude of all harmonics at full resolution [32].

A set of reference data of *H*′*_m_*(**r**) and *φ*′*_m_*(**r**) are acquired without any sample to provide a baseline. They are used to normalize the sample data in order to remove instrumental factors, such as defects in the grid. In the final results, the normalized attenuation of the zeroth order or DC peak, −ln[*H*_0_(**r**)/*H*′_0_(**r**)], is the conventional intensity attenuation. The attenuation of the *m*th harmonic peak includes contributions from x-ray scattering along the direction of that peak. These are quantified by −ln[*H_m_*(**r**)/*H*′*_m_*(**r**)]+ln[*H*_0_(**r**)/*H*′_0_(**r**)], which is the directional scattering, or dark-field image [10], [11], [33], [34]. The differential phase image *φ_m_*(**r**)−*φ*′*_m_*(**r**) measures the refractive deflection of the x-rays in the direction of the *m*th harmonic peak [21], [26].

The experimental setup (Fig. 1A) consists of a tungsten-target x-ray tube operating at 1 mA cathode current with a focal spot size of approximately 50 µm, an x-ray detector with a pixel size of 60 µm and a matrix size of 1024×1024, and a square absorption grid of 127 µm period placed in the x-ray beam. The detector is a Princeton Instruments SCX:4096 x-ray camera consisting of a CCD array coupled to a GdOS phosphor via a 1:1 fiber optic coupler. The CCD array of 4096×4096 15 µm pixels is binned to 1024×1024 to match the resolution of the phosphor. To correct for the detector's sensitivity pattern and dark baseline signal, a dark baseline image and a flat-field image are acquired beforehand. The dark baseline is subtracted from data images, which are then divided by the flat-field image. The x-ray tube voltage is set to 40 kVp to image the St. Johnswort stems and 60 kVp to image the mouse specimen.

In the system layout in Fig. 1A, several factors are considered in setting the distances between the components. One is that the grid has a nominal focusing distance of 76 cm (30 inches), meaning that the passing slots in the grid are aimed at a focal spot at that distance. The second is that the source-to-detector distance should not be too great in order to maintain the photon flux density on the detector. The third is that the minimal angle of x-ray refraction and scattering that can be detected with this method scales with the ratio of (fringe period)/(grid to detector distance) [10], [35]. With these factors in mind, the distance between the source and detector is set at 108 cm, with the imaged sample and the grid arranged equidistant in between. The grid is rotated around the beam axis by 18.4° relative to the horizontal, phase stepping axis. This angle satisfies tan(*θ*) = 1/3, such that in the 2D Fourier space the projections of the 3×3 harmonic peaks onto the phase stepping axis are evenly spaced (Fig. 1B). A phase stepping series has 14 images, which is more than the minimum of 9 in order to improve signal-to-noise ratio of the results. The exposure for each image is 60 sec.

The experiments employ motionless electromagnetic phase stepping (EPS) [25] (Fig. 1A), where a magnetic field is applied to the x-ray tube via a solenoid coil to deflect the source point of the beam. This results in opposite displacements of the projection images of the sample and the grid. Since the sample and the grid are at different distances to the source point, there is a relative movement between them. The EPS images are digitally moved back to re-align the projections of the sample, leaving the grid shadows to scan over the sample. This effectively synthesizes the phase stepping process without physical movements.

## Results

**Figure 2:**
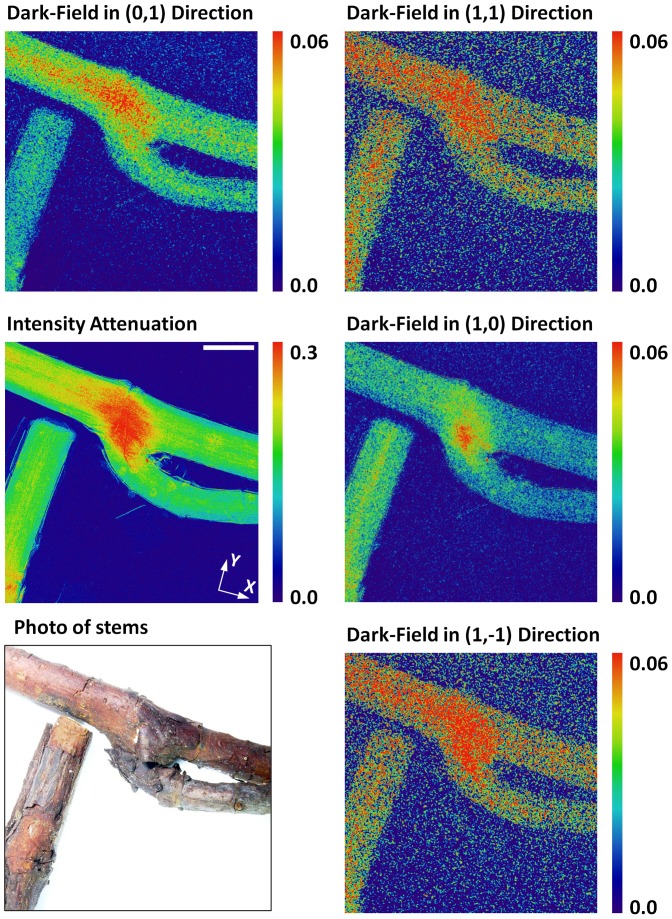
Imaging of x-ray scattering in St. Johnswort stems in multiple directions. The experimental setting is shown in Fig. 1A. A photograph of the stems is shown in the lower left. The mid-left image is the conventional intensity attenuation in negative log scale. The stems are arranged parallel with the X and Y axis of the absorption grid. The other images are dark-field images from the component harmonics of the 2D grid pattern, as defined in the main text. They measure the attenuation of the visibility of the pattern due to x-ray scattering in the sample. The mid-right image is the (1, 0) dark-field image, which measures scattering in the X direction. The top-left image is from the (0, 1) harmonic and measures scattering in the Y direction. The other two images measure scattering in the diagonal directions. The microscopic cellulose fibers of the stems are organized along their lengths, such that the x-rays are most strongly scattered perpendicular to the fibers and thus the axes of the stems. This gives rise to visibly different scattering signals from the same location between the (0, 1) and (1, 0) dark-field images. The anisotropy of x-ray scattering reflects the directional order of the composite structure of the stems. The orthogonal harmonic components have higher amplitudes than the diagonal ones, leading to higher signal-to-noise ratio in their dark-field images. Scale bar in mid-left image, 4 mm.

In a first demonstration, the stems of a St. Johnswort (*Hypericum calycinum*) plant are imaged in the arrangement of Fig. 1A. X-ray scattering by the ordered cellulose fibers of the stems is anisotropic and strongest in the plane perpendicular to the length of the stems. As a result, the fringes of the 2D grid pattern are blurred to various degrees dependent on how the stems are aligned with them. This effect is quantified by the scattering, or dark-field images, of the harmonics (Fig. 2). The relationship between the direction-dependent scattering signal and the underlying structural order is mathematically the same as that of the diffusion-weighted magnetic resonance image with the underlying diffusion tensor of water molecules.

**Figure 3:**
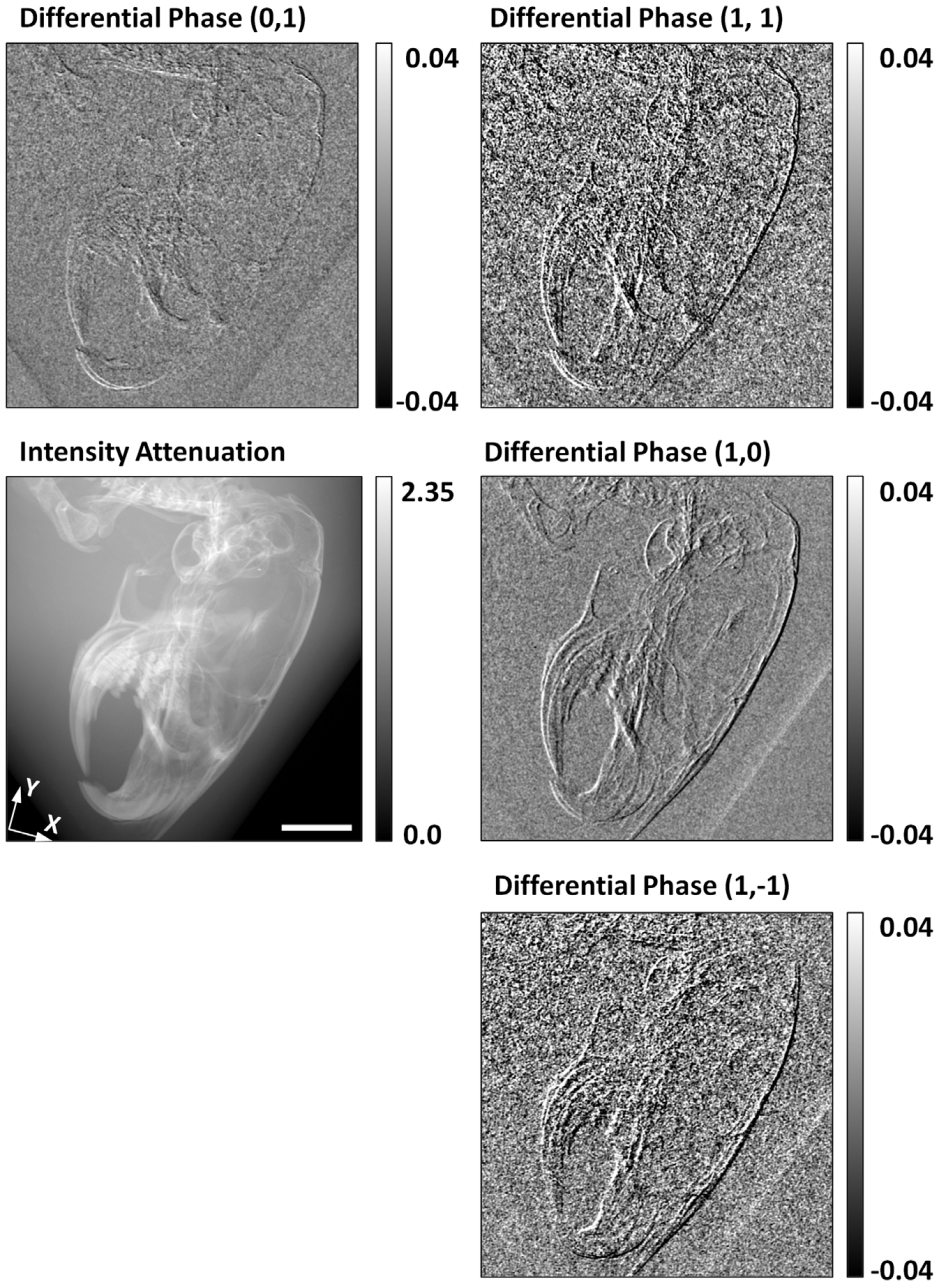
X-ray differential phase contrast images of the head region of a mouse specimen. The mid-left image is the conventional intensity attenuation in negative log scale. Other images are differential phase maps of the component harmonic waves of the 2D grid modulation. They measure the displacements of the lines in the grid pattern, the result of refractive bending of the x-ray wavefront. The differential phase map of the (1, 0) harmonic measures refractive bending along the X direction, while that of the (0, 1) harmonic is along the Y direction. The diagonal harmonics yield refraction information along those directions. Refractive bending occurs in areas of rapid transitions of the refractive index, such as bone-soft tissue interfaces. Thus, the differential phase images represent the gradient of the phase distribution of the wavefront in their respective directions. The harmonics highlight different segments of the interfaces, based on the alignment of the interfaces with the directions of the harmonic peaks. The orthogonal harmonics have higher amplitudes than the diagonal ones, yielding differential phase images of higher signal-to-noise ratio. Scale bar in mid-left image, 4 mm.

In another demonstration, the head region of a mouse specimen is imaged in the same arrangement. The mouse specimen was obtained in a protocol approved by the institutional Animal Care and Use Committee. A C57BL/6 wild-type 5 year old male mouse was euthanized and fixed in 10% buffered formalin solution for imaging experiments. The differential phase images of the various component harmonics of the 2D grid pattern are shown in Fig. 3. They quantify the refractive bending of the x-rays in the sample, or the equivalent gradient field of the wavefront phase distribution. Each harmonic is sensitive to the component of the gradient vector in its direction. Refraction is the strongest at interfaces of rapid transitions of the refractive index. It can be seen that the various harmonics highlight different segments of the bone-tissue interfaces based on their orientations.

## Discussion

Compared to 2D raster scanning, single-axis linear scanning simplifies the hardware instrumentation for wavefront measurements. It also enables electromagnetic phase stepping of 2D periodic patterns, in which the x-ray source is electronically scanned. EPS removes the need for mechanical movements and the associated stability and reproducibility issues. It also reduces hardware cost by replacing precision motor systems with a magnetic field coil. On the other hand, linear phase stepping of 2D patterns involves more computation. Specifically, the inverse Fourier transform in 2D raster scanning is replaced with a least-square fitting.

Linear phase stepping samples the sum of a number of oscillations of different frequencies along a single axis. To adequately capture and resolve the frequency components, both the sampling density and sampling range have certain requirements. The sampling density is represented by the step size *d* in real space, which should be sufficiently small to resolve the smallest oscillation period *P*_min_ along the scan axis, i.e., *d*<*P*_min_/2; the sampling range *D* should be large enough to resolve the smallest difference in the frequencies of the various oscillations Δ*f*_min_, i.e., *D*>1/Δ*f*_min_.

Generally, a square grid pattern with detectable harmonic amplitudes up to the *N*th order contains a (2*N*+1)×(2*N*+1) square matrix of harmonic peaks in the reciprocal space. By choosing a phase stepping scan axis at an angle *θ* =  arctan[1/(2*N*+1)] from the horizontal fringe line, the projections of the peaks onto the scan axis are evenly spaced. If we denote the spacing between adjacent projections as Δ*f*, the harmonics can be adequately sampled in real space by (2*N*+1)×(2*N*+1) points of uniform spacing of 1/(2NΔ*f*). This follows the rule that the minimum number of sampling points is equal to the number of 2D harmonic peaks to be determined. In practice, more sample points can be taken for better signal-to-noise ratio.

Beyond square and rectangular patterns, a generic 2D periodic pattern may not yield a rectangular lattice of harmonic peaks in the reciprocal space. As a result, their projections onto a scan axis are not evenly spaced. For a specific 2D modulation, non-uniform sampling steps may be considered in conjunction with the angle of the scan axis to optimize the stability of the inversion process from real space to the reciprocal space.
